# A Two-Stage Voting-Boosting Technique for Ensemble Learning in Social Network Sentiment Classification

**DOI:** 10.3390/e25040555

**Published:** 2023-03-24

**Authors:** Su Cui, Yiliang Han, Yifei Duan, Yu Li, Shuaishuai Zhu, Chaoyue Song

**Affiliations:** 1 Department of Electronic Information, Engineering University of Chinese People’s Armed Police Force, Xi’an 710086, China; cuisu3567@163.com (S.C.);; 2 Department of Computer and Information Technology, University of Pennsylvania, Philadelphia, PA 19019, USA

**Keywords:** sentiment classification, ensemble, concurrent, erroneous data, heterogeneous PDL, 2SVB

## Abstract

In recent years, social network sentiment classification has been extensively researched and applied in various fields, such as opinion monitoring, market analysis, and commodity feedback. The ensemble approach has achieved remarkable results in sentiment classification tasks due to its superior performance. The primary reason behind the success of ensemble methods is the enhanced diversity of the base classifiers. The boosting method employs a sequential ensemble structure to construct diverse data while also utilizing erroneous data by assigning higher weights to misclassified samples in the next training round. However, this method tends to use a sequential ensemble structure, resulting in a long computation time. Conversely, the voting method employs a concurrent ensemble structure to reduce computation time but neglects the utilization of erroneous data. To address this issue, this study combines the advantages of voting and boosting methods and proposes a new two-stage voting boosting (2SVB) concurrent ensemble learning method for social network sentiment classification. This novel method not only establishes a concurrent ensemble framework to decrease computation time but also optimizes the utilization of erroneous data and enhances ensemble performance. To optimize the utilization of erroneous data, a two-stage training approach is implemented. Stage-1 training is performed on the datasets by employing a 3-fold cross-segmentation approach. Stage-2 training is carried out on datasets that have been augmented with the erroneous data predicted by stage 1. To augment the diversity of base classifiers, the training stage employs five pre-trained deep learning (PDL) models with heterogeneous pre-training frameworks as base classifiers. To reduce the computation time, a two-stage concurrent ensemble framework was established. The experimental results demonstrate that the proposed method achieves an F1 score of 0.8942 on the coronavirus tweet sentiment dataset, surpassing other comparable ensemble methods.

## 1. Introduction

Twitter is a prevalent social media service platform that people use to express their opinions, experiences, and emotions [1]. There are numerous tweets with varying sentiments on Twitter. Analyzing the sentiment tendencies in users’ tweets is highly meaningful for social-network opinion analysis [2]. For instance, during the social-network opinion event triggered by the novel coronavirus pneumonia, any tweets with information about the outbreak will be emphasized and amplified due to the huge information gap between news information and the audience. Negative sentiment tweets can have a tremendous adverse impact on the public and society, and researchers, companies; and governments are increasingly paying attention to them [3,4]. The study of social network sentiment classification also has great practical value in various fields, such as public opinion detection [5], market-trend analysis [6], and product feedback analysis [7].

Social media sentiment classification represents a fast-evolving research domain within the field of natural language processing (NLP). Traditional sentiment classification approaches comprise both lexicon-based and corpus-based methodologies. The lexicon-based approach mandates the usage of an annotated sentiment lexicon [8] to determine the sentiment score of each text, which is subsequently utilized to evaluate the sentiment polarity and intensity of the text [9,10,11]. Conversely, the corpus-based approach involves the utilization of a massive manually annotated corpus as a dataset, followed by sentiment classification through a classifier. Early studies centered on the extraction of hand-designed features from text [12], which were subsequently used in machine learning models (ML), such as naive Bayes (NB) [13,14], k-nearest neighbor (KNN) [15,16], and support vector machine (SVM) [17,18] models, for sentiment classification. Recently, sentiment-classification methods based on deep learning (DL) models, such as convolutional neural networks (CNNs) [19], recurrent neural networks (RNNs) [20], long short-term memory (LSTM) [21], andthe gated recurrent unit (GRU) [22], have achieved remarkable results. The PDL models based on transformers [23] have been pre-trained on a large corpus of information and have significantly improved the accuracy of various NLP tasks. The PDL model obtained excellent performance in various sentiment-classification tasks, such as target-level sentiment classification [24], fine-grained sentiment classification [25], and aspect-level sentiment classification [26].

Ensemble learning can improve the performance of sentiment classification by combining multiple models to produce an optimized model [27]. As such, ensemble methods have been employed in numerous sentiment-classification tasks [28]. One of the main reasons behind the success of ensemble methods is the augmented diversity of underlying classifiers [29]. Generating diverse datasets from the original dataset enables the production of diverse base classifiers. The bagging [30] method utilizes the random sampling technique to construct diverse datasets. However, this method overlooks the utilization of erroneous data. To overcome this issue, the AdaBoost [31] method continually trains by assigning higher weights to misclassified samples and achieving a lower error rate. Nevertheless, AdaBoost implements the ensemble algorithm in a sequential manner, leading to a prolonged computation time. Concurrent structures can effectively reduce computation time. The voting [32] method constructs a concurrent ensemble framework to fuse diverse classifiers but disregards the diversity of input data. To leverage diverse datasets in a concurrent structure, the blending [33] and stacking [34] methods employ k-fold cross-segmentation to divide original data, constructing a two-layer network structure to enhance ensemble performance. However, current concurrent ensemble methods lack the utilization of erroneous data. In general, the current sequential ensemble methods utilize erroneous data but suffer from long computation times, whereas the concurrently structured ensemble methods boast fast computation times but lack the utilization of erroneous data.

To address this issue, this paper proposes a new concurrently structured ensemble method (2SVB). This method not only constructs concurrent ensemble structures to reduce computation time but also leverages erroneous data to enhance ensemble performance. Our research revolves around correctly identifying the sentiments linked to coronavirus tweets. We established a two-stage concurrent training framework and a two-stage ensemble method utilizing five heterogeneous pre-training frameworks of PDL models as base classifiers.

The main contributions of this paper are as follows:

- This paper proposes a novel ensemble method called 2SVB. The proposed method utilizes a two-stage data processing approach that not only generates diverse data but also effectively utilizes erroneous data.
- We utilize a base classifier group comprising five PDL models with heterogeneous pre-training frameworks to enhance diversity. The selected base classifier group outperforms other combinations in terms of performance.
- The proposed method uses two-stage concurrent training and an ensemble framework that allows for concurrent computation of all training processes except for the erroneous-data-collection process. We also propose a concurrent ensemble method of cascaded voting for the stage-2 ensemble, which enhances the diversity of concurrent ensemble algorithms.
- Compared to other ensemble methods, the 2SVB method demonstrates better performance. Our research has the potential to enhance the accuracy of various applications, such as sentiment analysis, rumor detection, and hate-speech classification.

The remainder of this paper is structured as follows. Section 2 provides an overview of related work on sentiment classification and ensemble methods. Section 3 outlines the proposed 2SVB framework and its various modules. Section 4 details the experiments conducted and the accompanying result analysis. Finally, Section 5 presents the concluding remarks.

## 2. Related Works

### 2.1. Sentiment Classification

Social network sentiment classification is a technique that has significant practical value and can solve the phenomenon of cluttered information in online comments to a certain extent. Sentiment classification is an important element in NLP text classification tasks, which often use corpus-based approaches. This method accomplishes sentiment classification by using a large manually labeled corpus as a dataset and employing a classifier such as a ML model. Pang et al. [35] were the first to utilize three machine learning methods (NB, ME, and SVM) for emotion classification. A SVM featuring bag-of-words was the most effective in the experiments. This idea has inspired many studies that focus on designing efficient features to improve sentiment-classification performance. In recent years, DL models have gained significant traction in social network emotion classification tasks. The RNN [36] and its extensions, such as Bi-LSTM [37], the gated recurrent neural network (GRNN) [38], and the adaptive recursive neural network (ARNN) [39], have demonstrated exceptional performance in sentiment-classification tasks. CNN [40] models have also exhibited promising results in classification tasks. Wang et al. [41] employed coarse-grained local features generated by CNN and long-range dependencies learned through RNN for sentiment analysis of short texts.

Transformer-based PDL models are pre-trained on a vast corpus of information to significantly enhance the accuracy of various NLP tasks. Most PDL models are classified into autoregressive language models, such as GPT (generative pre-trained transformer) [42] and ELMO (embedding from language model) [43], and autoencoder language models, such as BERT (bidirectional encoder representation from transformers) [44] and RoBERTa (robustly optimized BERT approach) [45]. Autoregressive models estimate the generative probability distribution of a string of text sequences and can compute text sequence probabilities in either a forward or backward direction. However, either modeling approach is unidirectional, and it is impossible to view both the left and right sides of a word when predicting that word. On the other hand, autoencoder language models reconstruct the original data from corrupted input text sequences, capturing information from both the left and right sides of the word when predicting the word. During the training process, the original sequence is reconstructed by predicting the words in the masked position. Generally, autoregressive models perform better on text generation tasks, whereas autoencoder models perform better on language comprehension tasks. Abdelgwad et al. [46] proposed an aspect-level sentiment analysis method based on BERT for the Arabic sentiment-polarity classification task and achieved good results. Choudrie et al. [47] developed a multi-class sentiment classifier system based on RoBERTa and transfer learning, applied to the study of sentiment analysis of COVID-19.

### 2.2. Ensemble Methods for Sentiment Classification

The ensemble method enhances the performances of individual classification models and reduces the variance of predictions by fusing multiple base classifiers to create an optimized classifier [48]. This technique is designed to stabilize accuracies and improve the robustness and generalizability of the final model.

**Voting** [32]: This technique produces concurrent ensemble networks using heterogeneous base classifiers. There exist popular voting methods such as majority voting [49] and weighted averaging [50] algorithms. The weighted averaging algorithm computes the weighted average of the probability values of each classifier classification and selects the label with the highest probability value. Tang et al. [51] proposed an ensemble network for detecting chest X-ray images of COVID-19 cases based on the weighted averaging technique. The majority voting algorithm aggregates the labels of all classifiers of the same category and selects the label of the category with the most votes. Malla et al. [52] used a majority voting method to fuse three PDL models, namely, RoBERTa, CTBERT, and BERTTweet, for the detection of informative COVID-19 tweets and achieved excellent performance.

**Bagging** [53]: It is a sequential ensemble network that constructs diverse classifiers by inputting dissimilar datasets. This technique produces sequential ensemble networks generating diverse classifiers by using dissimilar datasets. The bagging method achieves the desired dataset by employing N random samples. Subsequently, N predictors are independently trained for each of the randomly sampled sets. The predictor predictions are then subjected to the ensemble strategy to derive the final results. Random subspaces [54] and random forests [55] are the most commonly utilized models that use bagging methods. Risch et al. [56] proposed a bagging ensemble network constructed using multiple fine-tuned BERT models. It was ascertained that randomly sampled datasets can achieve better performance in PDL-based ensemble methods.

**Boosting** [57]: It is a sequential ensemble network that improves ensemble performance by valuing the wrong data from previous classifiers. Initially, each data point in the dataset is assigned the same weight. Data that are classified incorrectly in model N are given higher weights in model N+1. Each classifier endeavors to improve the stability of the entire process by reducing the error of the previous classifier. There are commonly used boosting methods, such as AdaBoost [58], and XGBoost [59]. Mewada et al. [60] proposed a method based on synthetic attention in bidirectional encoder representations from transformers (SA-BERT), an XGBoost ensemble method for aspect-level sentiment analysis, and obtained extraordinary results.

**Stacking** [34]: It divides the dataset by using the N-fold stacking technique and has a two-layer stacked network. Firstly, the training set is partitioned into k subsets, and the base classifier is trained in the initial k-1 subsets and predicted in the kth subset. This process is repeated until each subset is predicted. The predictions from the training set are then utilized as features for constructing the second layer and modeling predictions using algorithms such as logistic regression (LR). Rao et al. [61] proposed a stacking network based on a co-attentive mechanism, which uses sentiment comments and sequential comments as auxiliary data to achieve good performance.

**Blending** [33]: This method has a similar two-layer network structure as stacking, but the data-division approach is different. The blending technique splits the training set into two parts. Firstly, the base learners of the first layer train in the initial part of the training set. Subsequently, the second part of the training set and the prediction outcomes of the first layer form a new training set, which is fed into the second layer model for the final prediction. Liu et al. [62] developed an ensemble network incorporating blending and stacking methods for fake news classification, which yielded excellent results.

## 3. The 2SVB

### 3.1. Framework

**Figure 1:**
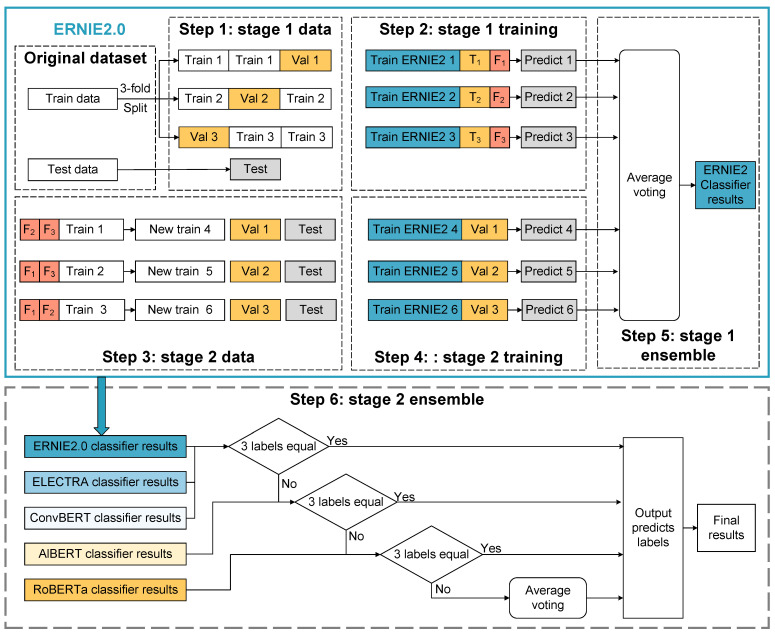
The framework of the 2SVB approach.

Herein, the proposed 2SVB integration method is presented for use in the coronavirus tweets sentiment classification task. It consists of three main parts: data processing, training, and ensemble. Each part is divided into two stages, and the main process includes six steps, as shown in Figure 1.

Step 1: Stage-1 data processing. The coronavirus tweet sentiment dataset was divided into three groups of datasets according to the 3-fold cross-segmentation method.

Step 2: Stage-1 training. Base classifiers were trained on the three sets of data to obtain three classifiers. The classifiers’ output results and the validation-set-predicted erroneous data were saved after making predictions with the validation set and the test set.

Step 3: Stage-2 data processing. The erroneous data from the validation set were utilized to update the stage-1 training set data. This process generated three new datasets while keeping the validation set and the test set unchanged.

Step 4: Stage-2 training. After generating the new datasets, base classifiers were trained using the three new training sets. The classifiers were then validated using the validation set, and the predictions were made on the test set data. Finally, the predictions were saved for further use.

Step 5: Stage-1 ensemble. The prediction results obtained from the six classifiers trained in stage 1 and stage 2 were integrated using an average voting algorithm.

Step 6: Stage-2 ensemble. The output results obtained from the five stage-1 ensemble classifier groups were integrated using the cascade voting method.

### 3.2. Data Processing

To better utilize the erroneous data, we considered a two-stage data processing approach. In this approach, we utilized a 3-fold cross-segmentation approach for stage 1 and an erroneous data-supplementation approach for stage 2.

#### 3.2.1. Stage-1 Data Processing

For stage-1 data processing, we used the 3-fold cross-segmentation method to split the coronavirus sentiment data training set into datasets. Firstly, the training set was divided into K subsets. Then, K-1 copies of these subsets were used as the training set, and the remaining copy was used as the validation set. This process was repeated until each subset was predicted, and the coronavirus tweet sentiment data’s test set was used as the test set to obtain K datasets. The greater the value of K, the more reliable the average error was considered as a generalization error. However, the corresponding computational cost would increase linearly. Due to the high time complexity of PDL models, we chose a K of 3 for this study.

Since the validation and test sets were not involved in training, the traditional division using 3:1:1 or other ratios would result in a large amount of data that could not be applied for learning. Stage-1 data processing could make better use of the training set data. All samples in the training set were bound to become training data and also bound to have the opportunity to become the validation set once. When the ensemble method was used, the variability of the training data enabled the base classifiers to learn dissimilar content. This could provide better results when using the ensemble algorithm.

#### 3.2.2. Stage-2 Data Processing

The objective of stage-2 data processing was to supplement the stage-1 training with erroneous data. To achieve this, we cross-supplemented the incorrect data from the validation set to the training set, making the incorrect data present twice in the training set. This allowed the model to train on more varied data and learn more about data that are harder to predict correctly. Specifically, after training in stage 1, each validation subset was categorized into two groups, true data (True) and false data (False). The training data were then updated using three sets of predicted false validation data (False1, False2, False3). The false validation data were added to the other two training sets separately to generate three new training sets. The validation set and test set followed the stage-1 dataset. As a result, three new datasets were generated.

**Figure 2:**
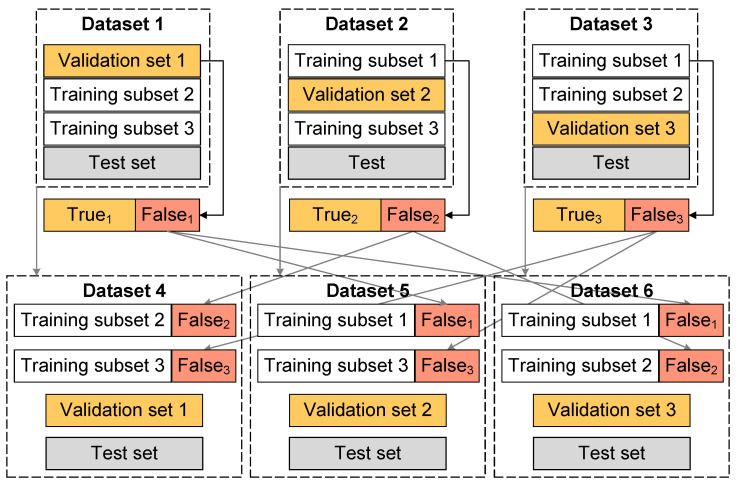
Stage-2 data processing.

The two-stage data processing approach is depicted in Figure 2. The training set was split into three subsets using the 3-fold cross-segmentation approach. Training subset 1 was utilized as the validation set for dataset 1, training subset 2 was used for the validation set of dataset 2, and training subset 3 was used for the validation set of dataset 3. In stage-1 training, validation sets 1, 2, and 3 were categorized into true data (True1, True2, True3) and false data (False1, False2, False3), respectively. In stage-2 data processing, for instance, in dataset 4, training subset 2 added the erroneous data from validation set 2, training set 3 added the erroneous data from validation subset 3, and the validation set continued to use training subset 1. As the erroneous data in validation set 2 were from training subset 2, the erroneous data were increased in training subset 2. Therefore, the amounts of harder-to-predict data (erroneous data) in the training sets of datasets 4, 5, and 6 were doubled. This implied that the data added in stage 2 would not be present in the validation and test sets.

### 3.3. Training

During the training stage, we utilized the PDL model as the base classifier, which is a language-representation model. The model was founded on a transformer, which is a stack of multiple transformer encoders, and utilized an enormous volume of unlabeled data to pre-train a generic “language understanding” model through unsupervised methods. Subsequently, the pre-trained model was fine-tuned to execute the desired NLP task. To augment the diversity of base classifiers, we employed five PDL models based on the heterogeneous pre-training framework.

#### 3.3.1. Base Classifiers

**RoBERTa** [45] is an enhanced version of BERT. Several improvements have been made on the BERT pre-training framework for better processing of natural language tasks. RoBERTa uses a masked language model (MLM) task based on a dynamic masking strategy. By randomly masking some words in the text and then asking the model to predict the masked words, the model’s language comprehension is improved. The model is also pre-trained with a larger number of model parameters, a larger batch size, and more training data. The version of RoBERTa implemented in this paper was “roberta-base”, which encompasses 12 encoder layers, 768 hidden units, 12 attention heads, and 101 million parameters.

**ERNIE2** [63] (enhanced language representation with informative entities) has a pre-training framework based on multi-task learning and continuous training. Multi-task learning incorporates three key types of pre-training tasks: word-aware pre-training tasks, structure-aware pre-training tasks, and semantics-aware pre-training tasks. The continuous training process is categorized into two steps: building unsupervised pre-training tasks and incrementally updating the model through multi-task learning. The version of ERNIE2 implemented in this paper was “ernie-2.0-base-en”, which encompasses 12 encoder layers, 768 hidden units, 12 attention heads, and 103 million parameters.

**ELECTRA** [64] (efficiently learning an encoder that classifies token replacements accurately) has a new pre-training framework combining a generator and a discriminator. The generative MLM pre-training task was changed to a discriminative replaced token detection (RTD) task to determine whether the current token has been replaced by a language model. The generator module adopts the classical MLM approach of BERT, which makes the text masked randomly. The role of the discriminator module is to distinguish whether each input token is the original one or the replaced one. By adding up the losses of the two modules, the learning difficulty of the discriminator is gradually increased, and plausible tokens can be learned. ELECTRA discards the generator and uses only the discriminator in the fine-tuning phase, and we used a version of “electra-base” with 12 discriminator layers, 768 hidden cells, 12 attention headers, and 109 million parameters.

**ConvBERT** [65] (improving BERT with span-based dynamic convolution) integrates convolution into self-attention to form a new pre-training framework based on a hybrid attention mechanism. ConvBERT uses span-based dynamic convolution to replace multi-head attention with model local dependencies. Multi-head attention can extract overall features, and span-based dynamic convolution can extract local features. ConvBERT combines the advantages of both and is the first to propose combining convolution to improve BERT efficiency. The version of ConvBERT used in this paper was “convbert-base”, having 12 encoder layers, 768 hidden units, 12 attention heads, and 106 million parameters.

**AlBERT** [66] (a lite BERT for self-supervised learning of language representations) significantly reduces the number of pre-trained model parameters and builds a pre-training framework with fewer parameters. There are three main improvements: factorization of the embedded parameters through matrix decomposition, sharing of parameters between layers through cross-layer parameter sharing, and changing the next statement prediction (NSP) task of the BERT pre-training framework to sentence order prediction (SOP). In general, AlBERT constructs a pre-training framework that reuses one encoder to reduce the number of parameters. The version of AlBERT used in this paper was “albert-base-v2” with 1 encoder layer, 12 repeating layers, 768 hidden units, 12 attention heads, and 12 million parameters.

#### 3.3.2. Stage-1 Training

In stage-1 training, the training set from stage-1 data processing was utilized for training, the validation set for validation, and the test set for predicting outcomes and preserving the erroneous data from the validation set. The same procedure was carried out for all PDL models. To elaborate, let us consider the example of the ERNIE2 model. ERNIE2 underwent training using three sets of stage-1 training and was validated using the validation set to obtain three classifiers. Subsequently, each classifier was tested against the test set, and the results from the test set and the data of prediction errors from the validation set were recorded. The stage-1 training of each base classifier can be computed concurrently.

#### 3.3.3. Stage-2 Training

In stage-2 training, the training set from stage-2 data processing was utilized for training, the validation set for validation, and the test set for predicting outcomes. The same procedures were executed for all PDL models as in the stage-1 training. Take the ERNIE2 model as an example. It was trained using three fresh training sets from stage-2 data processing and validated using the validation set, and three new classifiers were obtained. The test set’s data were then predicted, and the prediction results of each classifier were saved. The prediction results of the test sets trained in stage 1 and stage 2 are reported in Table 1. RoBERTa resulted in the lowest average evaluation metrics, whereas ERNIE resulted in the highest average evaluation metrics. The stage-2 training of each base classifier can be computed concurrently.

### 3.4. Ensemble

To effectively integrate the diverse base classifiers, we adopted a two-stage ensemble strategy. The stage-1 ensemble was a local fusion stage that involved integrating the predictions made by the classifiers utilizing an average voting algorithm to identify the sentiment polarity from the original tweet data. The stage-2 ensemble was a global fusion stage. A cascaded voting algorithm was devised to further integrate all classifier groups by enhancing the majority voting and average voting methods.

#### 3.4.1. Ensemble Methods

**Average voting**: It is a special weighted average method where all classifiers have the same weights, which is also known as the soft voting algorithm. This algorithm is used to aggregate prediction probabilities from multiple base classifiers and selects the class labels that are most likely to be predicted. To achieve this, the output of each fine-tuned PDL model is connected to a linear layer and a softmax function for classification, which produces a classification label *j* and its corresponding probability *p*. The probability values for each class label are then summed and averaged, and the label with the highest probability is selected as the prediction result. The average voting method is calculated as shown in Equation (1).

(1) $${\hat{y}}_{s}=\frac{1}{n}\sum_{i=1}^{n}p_{i,j},$$

where $p_{i,j}$ denotes the probability of class label *j* being predicted by the *i*-th classifier (out of *n* classifiers).

**Cascade voting**: We propose the cascade voting algorithm, which improves upon the majority voting and weighted average algorithms. This algorithm was designed to output confident prediction labels by increasing the number of classifiers until a confident prediction could be made based on the current set of classifiers. Specifically, if the prediction labels of three classifier groups were the same, this prediction label was considered confident and could be output directly. However, if the labels were not identical, then the number of classifiers was increased until there were three predicted identical labels. If there were no three identical prediction labels for five classifiers, the average voting algorithm was used to output the final prediction. The specific process of the cascade voting algorithm is shown in Algorithm 1.
**Algorithm 1** Cascade voting.**Input:** $T_{k}$, Classifier *i***Output:** Test sets with labels (*j*)1:$l_{\left(i,j\right)}$: Classifier *i* predicted labels *j* (Neutral 0, Positive 1, Extremely positive 2, Negative 3, Extremely negative 4).2:$p_{\left(i,j\right)}$: Classifier *i* predicts the probability of label *j* (Neutral 0 1, Positive 0 1, Extremely positive 0 1, Negative 0 1, Extremely negative 0 1).3:$T_{k}$: The kth sample of the test set.4:**for** k = 0 to N **do**5:   **if** [($l_{\left(1,j\right)}$), ($l_{\left(2,j\right)}$), ($l_{\left(3,j\right)}$)] have 3 same labels *j* **then**6:     $T_{k}\leftarrow j$;7:   **else if** [($l_{\left(1,j\right)}$), ($l_{\left(2,j\right)}$), ($l_{\left(3,j\right)}$), ($l_{\left(4,j\right)}$)] have 3 same labels *j* **then**8:     $T_{k}\leftarrow j$;9:   **else if** [($l_{\left(1,j\right)}$), ($l_{\left(2,j\right)}$), ($l_{\left(3,j\right)}$), ($l_{\left(4,j\right)}$), ($l_{\left(5,j\right)}$)] have 3 same labels *j* **then**10:     $T_{k}\leftarrow j$;11:   **else**12:     Max ($\sum_{i=1}^{5}p_{i,0}$, $\sum_{i=1}^{5}p_{i,1}$, $\sum_{i=1}^{5}p_{i,2}$, $\sum_{i=1}^{5}p_{i,3}$, $\sum_{i=1}^{5}p_{i,4}$) corresponds to label *j*;13:     $T_{k}\leftarrow j$;14:   **end if**15:**end for**

#### 3.4.2. Stage-1 Ensemble

The stage-1 ensemble was a local fusion stage, where each PDL model from stage-1 and stage-2 training produced six classifiers that formed a classifier group. These classifier groups were integrated using the average voting ensemble method. Specifically, stage-1 training was conducted on the 3-fold cross-segmentation dataset to obtain classifiers 1, 2, and 3; and stage-2 training was conducted on an incorrectly updated dataset to obtain classifiers 4, 5, and 6. As shown in Table 1, most of the classifiers had higher accuracy after stage-2 training than during stage 1. However, due to the high variance present in the PDL model, not all stage-2 classifiers were more accurate than their stage 1 counterparts. When classifiers 1, 2, and 3 made predictions on the test set, the probability of correct prediction for the harder-to-predict samples was low. After additional learning and training of the harder-to-predict data during stage-2 training, the probability of correct predictions for these samples improved. To achieve higher ensemble performance, we integrated the prediction results of classifiers obtained from the training of six dissimilar datasets using the average voting algorithm. Some of the harder-to-predict data had smaller probabilities of receiving correct labels from classifiers of stage-1 training, but higher probabilities of receiving correct labels from classifiers with stage-2 training. By summing up the probability values for some of the harder-to-predict data through the average voting algorithm, the probability of correct labels for these data was increased, and previously existing incorrect data were corrected, achieving improved accuracy.

#### 3.4.3. Stage-2 Ensemble

**Figure 3:**
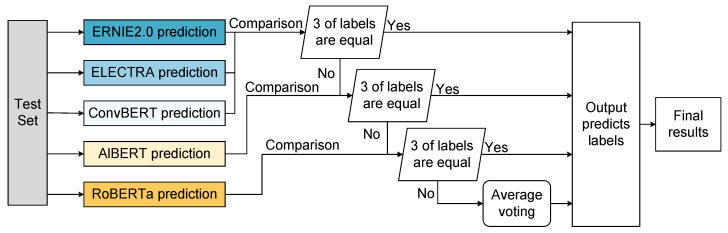
Stage-2 ensemble process.

In the stage-2 ensemble, we employed the cascade voting ensemble method, which is illustrated in Figure 3. Firstly, the labels predicted by the ERNIE2, ELECTRA, and ConvBERT classifier groups were compared. If there were three labels that were equal, the label was output immediately. If not, the AlBERT classifier group was added for comparison. When three of the four classifiers had the same label, the label was output. If not, the RoBERTa classifier group was added for comparison. When three of the five classifiers had the same label, the label was output. If none of the above conditions were met, the average voting algorithm was applied to the label probabilities output by the five classifiers, and the label with the highest probability value was output. The integration order was based on the F1 scores of the PDL model for the stage-1 ensemble, which was arranged in descending order as ERNIE2, ELECTRA, ConvBERT, AlBERT, and RoBERTa. This order was chosen to allow as many sample labels as possible to output predictions using just three classifiers when the cascade voting strategy was used for the stage-2 ensemble. As shown in Table 2, the checkmark (✓) represents the classifier used. When classifier group 4 was selected, the number of predicted samples with only one type of label was 3372. When classifier groups 1–3 were selected, the number of predicted samples with only one type of label was always less than that of classifier group 4.

We created a cascade voting method, which combines the majority voting and average voting algorithms, and the base classifier group we used was five. When three of the five classifier groups had the same predicted result, this result could be considered confidential in the current state based on the majority voting principle. However, since most of the data had the same predicted results, not all of them needed to undergo the majority voting process. As shown in Table 2, classifier group 4 had 3372 samples with only 1 predicted label, 413 samples with 2 predicted labels, and 13 samples with 3 predicted labels from the 3 base classifiers. Classifier group 5 had 3183 samples with 1 predicted label, 588 samples with 2 predicted labels, only 27 samples with 3 predicted labels, and no samples with 4 predicted labels. Similarly, classifier group 6 had 3045 samples with 1 predicted label, 715 samples with 2 predicted labels, only 38 samples with 3 predicted labels, and no samples with 4 or 5 predicted labels. Therefore, most samples could obtain confident results using 3–4 base classifier groups. Samples with two predicted labels had at least three predicted values that were the same for the five base classifiers, so samples with 1 label and 2 labels could obtain confident results quickly with groups of 3–5 classifiers. Only a small number of samples had three different predicted labels, which are often more difficult to predict accurately. When five classifier groups had three predicted labels, the label distribution was likely to be 2:2:1, which could not yield a confident result using the majority voting principle. To address this issue, the cascade voting method used the average voting algorithm to output results when there were no three identical labels in the five classifier groups. Thus, the prediction time could be reduced to some extent by the cascade algorithm. Although the advantage of our prediction time in sentiment classification was not obvious with only 3798 prediction data, we believe that our method could have an advantage when applied to the sentiment classification of massive tweet information in social networks.

## 4. Experiments and Analysis

### 4.1. Dataset

The experiments in this paper also used the coronavirus tweet sentiment NLP text-classification dataset, which was published by data scientist Aman Miglani on the Kaggle competition platform. The dataset comprises tweets extracted from Twitter from 2 March to 14 April 2020 and contains users’ tweets with the following topic labels: coronavirus, coronavirus outbreak, coronavirus Pandemic, COVID-19. From about 17 March, the dataset also included the following additional hashtags: epitwitter, ihavecorona. The coronavirus tweet sentiment dataset was split into a training set and a test set, and Table 3 depicts the fundamental statistical data of the dataset.

**Figure 4:**
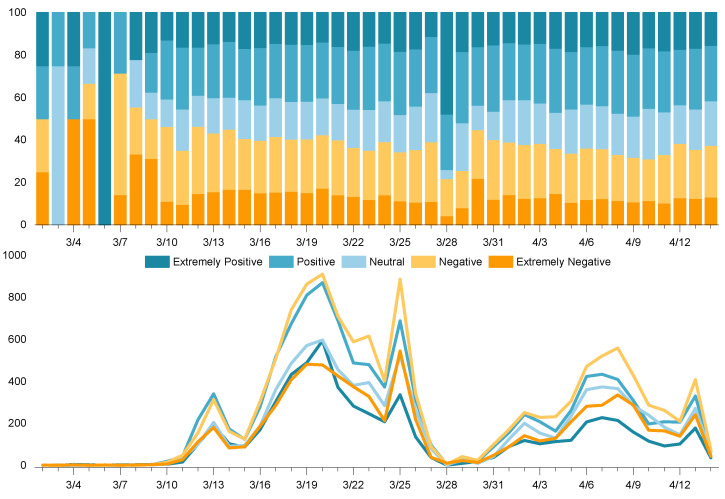
Daily sentiment category information of the coronavirus tweet dataset.

The dataset contained a total of 44,955 tweets, which were manually labeled with one sentiment label for each tweet by a data scientist. The labels are extremely positive, positive, neutral, negative, and extremely negative. The daily sentiment category information of the coronavirus tweet dataset is illustrated in Figure 4, which demonstrates that there were only a few tweets before March 11. Subsequently, there was a surge in coronavirus sentiment tweets from March 17 to March 26, followed by a low point from March 28 to March 30 and a slow increase thereafter. The dataset has many positive and negative tweets, and relatively fewer extremely positive and extremely negative tweets. The number of sentiment tweets was generally balanced across categories; no significant disparities were observed. Overall, the dataset provided a good basis for evaluating the performance of the proposed sentiment-classification method.

### 4.2. Baseline Models and Ensemble Approaches

Herein, the baseline models and the ensemble methods for comparison are presented. In the baseline ensemble methods, we used experiments performed with the best number of base classifiers presented in the original paper.

**Baseline models**

- SVM: A machine learning model based on support vector machines for text classification.
- Embedding: A basic embedding network used for text classification.
- 1-D Conv [67]: A 1-D convolutional network is used to process the embedding matrix and filter the embedding matrix of the whole sentence, extract some basic features from the larger embedding matrix, and compress them into a smaller matrix.
- Bi-LSTM [68]: A special kind of bidirectional recurrent neural network that can analyze the input using time series. It can better capture the semantic dependencies in both directions more efficiently.
- GPT2: An autoregressive language model built on the transformer decoder. A unidirectional language model was built using the transformer architecture of the decoder only.
- BERT: An autoencoder language model built on the transformer encoder. A multi-layer transformer encoder structure is used to build the entire model, resulting in a deep bi-directional language representation that incorporates left and right contextual information.
- XLNet: An autoregressive language model based on transformer-XL. The autoregressive structure is used to achieve bidirectional encoding.

**Ensemble approaches**

- Bagging [56]: A sequential ensemble network consisting of 15 BERT models. The method involves obtaining 15 datasets through random sampling and training 15 classifiers independently using the BERT models based on each of the randomly sampled sets. Ultimately, the prediction results are aggregated using an average voting algorithm.
- Boosting [69]: A sequential ensemble network consisting of nine BERT models. Initially, the first base classifier was trained to compute the prediction erroneous data and update the dataset’s weights. Specifically, the weights of the misclassified data were augmented, and the weights of the correctly classified data were reduced. Subsequently, multiple base classifiers were retrained, and the process of weight updating was repeated. Finally, the class labels were predicted using a fusion network.
- Stacking [70]: A network that applies the stacking strategy to the inside of BERT. The method constructs stacking networks that transfer knowledge from shallow models to deep models, and then progressively applies stacking to accelerate BERT training.
- Blending–stacking [62]: A concurrent ensemble framework that fuses blending and stacking networks. The method involves using 25 BERTs as the base classifier to partition the dataset for independent training based on the blending method. Then, six classifiers (three SVMs, LR, KNN, and NB) based on a 5-fold stacking technique were used for training and prediction. Finally, the LR method was used to avoid overfitting based on 5-fold cross-validation.
- Majority voting [52]: A concurrent ensemble network based on the majority voting algorithm. The base classifier of the network comprised five RoBERTa, five ERNIE2, five ELECTRA, five ConvBERT, and five AlBERT PDL models.
- Average voting [51]: A concurrent ensemble network based on the average voting algorithm. The base classifier of the network comprised five RoBERTa, five ERNIE2, five ELECTRA, five ConvBERT, and five AlBERT PDL models.
- 2SVB: Our proposed ensemble method.

### 4.3. Performance Measures

Four evaluation metrics were used to provide a comprehensive evaluation of the performances of the ensemble methods. They help us to understand the strengths and limitations of these models when making predictions in new situations.

*TP* stands for a true positive, which the model predicts as positive and is actually positive; *FP* stands for a false positive, which the model predicts as positive and is actually negative; *FN* stands for a false negative, which the model predicts as negative and is actually positive; and *TN* stands for a true negative, which the model predicts as negative and is actually negative.

Accuracy (*Acc*) is the ratio of the number of correctly predicted samples to the number of total predicted samples, as shown in Equation (2).

(2) $$Acc=\frac{TP+TN}{TP+TN+FP+FN}$$

Precision (*Pre*) is the ratio of the number of correctly predicted positive samples to the number of predicted positive samples, as shown in Equation (3).

(3) $$Pre=\frac{TP}{TP+FP}$$

Recall (*Rec*) is the ratio of the number of correctly predicted positive samples to the total number of actual positive samples, as shown in Equation (4).

(4) $$Rec=\frac{TP}{TP+FN}$$

F1 score (*F1*) combines the output results of precision and recall, as shown in Equation (5).

(5) $$F1=\frac{2Pre*\mathrm{Rec}}{Pre+\mathrm{Rec}}$$

### 4.4. Experimental Settings

All the experiments in this study were performed via the Baidu AI Studio interface on the Microsoft Edge browser with the following configuration: the server was configured with a 4-Cores CPU, a Tesla V100 GPU, 32 GB RAM, and 32 GB of video memory. The machine learning platform employed in the experiments was PaddlePaddle 2.4.0 with Python 3.7.

For the experiments, the SVM model used was “linersvc” from the sklearn toolkit. The hyperparameters of the DL models were set as indicated in Table 4. In the embedding method, an average pooling layer was used with an optimizer of Adam, an initial learning rate (initial lr) of $1\times 10^{-3}$, a batch size of 64, a maximum text length (max len) of 256, and a Relu activation function. In the 1-D Conv method, a convolutional layer and an average pooling layer were used with an optimizer of Adam. In the Bi-LSTM method, two forward and backward LSTM layers were used with an Adam optimizer. In the PDL model, a max len of 256, a batch size of 64, an initial lr of $5\times 10^{-5}$, and an AdamW optimizer were used. During training, the epoch was set to 10, and the currently trained model was evaluated every 100 steps. The current best model parameters and the vocabulary of the tokenizer were saved. In the bagging, boosting, stacking, and blending homogeneous ensemble methods, the same number of BERTs as in the original text were used as the base classifiers. RoBERTa, ERNIE2, ELECTRA, ConvBERT, and AlBERT were used as the base models in the majority voting and average voting ensemble methods, and each model was initially trained six times. In this paper, the experiments were not precisely tuned but trained with uniform hyperparameters. Better results may be obtained if exact parametrization is performed.

### 4.5. Comparison of Baseline Classifiers and Classifier Groups

The choice of the base model has a significant impact on the overall prediction performance of the ensemble method. In this study, we compared the performance when using different base classifiers and the performances of classifier groups based on the average voting algorithm.

#### 4.5.1. Performance Metrics of Baseline Classifiers

**Figure 5:**
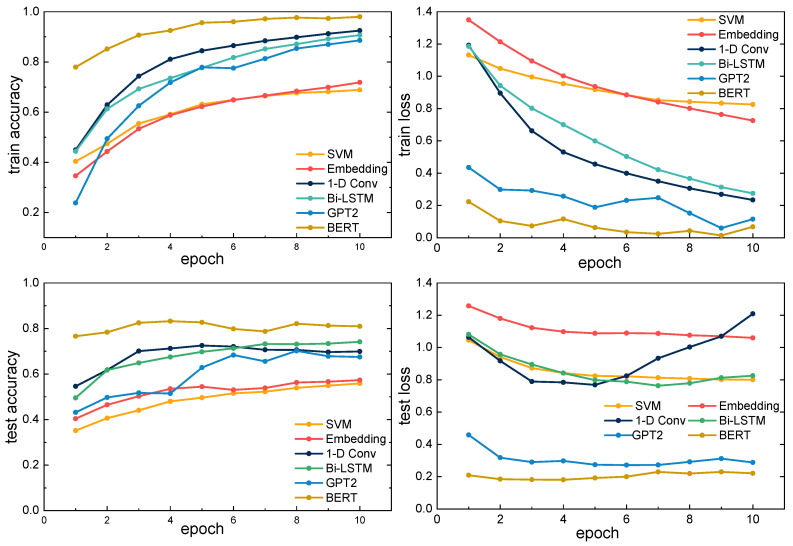
Comparison of training and testing process metrics of different models.

As shown in Figure 5, the SVM model resulted in the lowest training and testing accuracies and higher training and testing losses. The accuracies of the embedding and SVM models were similar, whereas the 1-D Conv and Bi-LSTM approaches resulted in higher training and testing accuracies. This indicates that the DL model performed better. Although the training accuracy of the 1-D Conv model was observed to be increasing and the training loss decreasing, the test accuracy decreased from the fifth epoch, and the test loss increased from the fifth epoch also, due to the over-fitting phenomenon due to 1-D Conv over-learning. GPT2 and BERT are both PDL models—BERT being an autoencoder language model and GPT2 an autoregressive language model. BERT had the highest training and test accuracy and the lowest training and test loss. Though GPT2 followed BERT in training and test loss, both its training accuracy and test accuracy were lower than those of 1-D Conv and Bi-LSTM. This suggests that the autoregressive feature of the PDL model of GPT2 is less effective than the autoencoder model of BERT in the text classification task.

**Figure 6:**
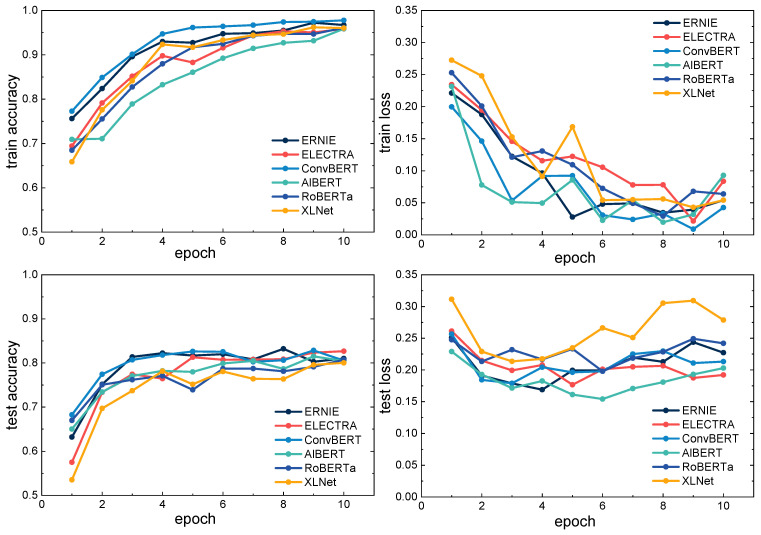
Comparison of training and testing process metrics of different PDL models.

Figure 6 shows the training and testing process of the PDL model. The monotonic curves of a few individual models exhibit anomalous behavior. This is due to the fact that in our experiment, all PDL models were not meticulously tuned, and the initial learning rate was uniformly set to $5\times 10^{-5}$. During the training of XLNet, the training loss suddenly increased in the fifth epoch. This learning rate was not suitable for the XLNet model, resulting in fluctuations in training up to the fifth epoch. Then, it started to converge again from the sixth epoch. This was since the PDL model used the AdamW optimizer to automatically adjust the learning rate for better convergence of the training process. ConvBERT obtained the highest accuracy and lowest loss during training, and AlBERT obtained the lowest accuracy and lowest loss. However, ELECTRA had the highest test accuracy and the lowest test loss during testing. XLNet had the lowest test accuracy and the highest test loss. The experiments showed that most PDL models for sentiment classification have good performance on the coronavirus tweet sentiment dataset.

#### 4.5.2. Performance Metrics of Classifier Groups

Here, the performances of the classifier groups formed by the average voting strategy are compared. As shown in Table 5, classifier group 1 had the worst performance, including an F1 score of 0.6455, which was lower than the F1 score of the base classifier Bi-LSTM (0.7417). Classifier groups 2 and 3 had F1 scores of 0.8089 and 0.7546, both lower than the F1 score of BERT (0.8325). The first three experimental groups yielded poor performance when the base classifiers were integrated when their performances varied widely. Classifier group 4 (0.8517) had a lower F1 score than classifier group 5 (0.8643). This showed that using a PDL model with better performance (ERNIE) integrated better than a model with poorer performance (GPT2). The F1 score for classifier group 6 was higher than that of classifier group 5. The BERT and RoBERTa models were more similar in structure, so the models with dissimilar structures had better ensemble performances. Classifier groups 7 and 8 had higher F1 scores than classifier group 6 after increasing the number of base classifiers. This indicates that increasing the number of classifiers could improve the model performance. However, the F1 score (0.8677) decreased when classifier group 9 continued to add XLNet on top of classifier group 8. This showed that if adding a new base classifier results in worse performance than the existing average, their performance would decrease. The experimental results showed that it is important to select the best members of the component predictors in the ensemble learning strategy. In the ensemble approach of PDL models, using models with larger structural differences as base classifiers could improve the performance of sentiment classification.

### 4.6. Performance Metrics for Different Ensemble Methods

**Figure 7:**
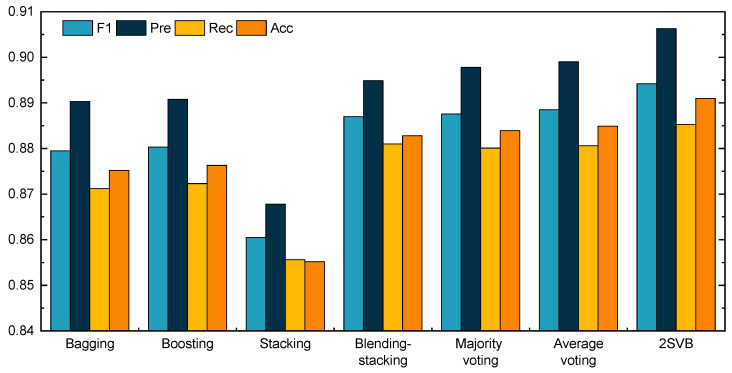
Performance metrics of different ensemble methods.

We compared the proposed method with six classical ensemble methods. As shown in Figure 7, the first three were sequential ensemble methods and the last four were concurrent ensemble methods. The bagging approach used 15 BERT models for the ensemble. It achieved an F1 score of 0.8795. The boosting approach used 9 BERTs as base classifiers and achieved a higher F1 score (0.8803) than the bagging method. The stacking approach integrated the BERT model from a shallow model into a deep model step by step. The F1 score of the stacking approach (0.8605) was lower than those of the bagging and boosting methods. The blending–stacking approach used 25 BERT-based classifiers and a 5-fold cross-validated stacking network. The experimental results show that the blending–stacking ensemble network structure was able to obtain better ensemble results.

Majority voting, weighted voting, and 2SVB used the same number of heterogeneous base classifiers. The results of experiments showed that the latter three heterogeneous ensemble methods perform better than the first four homogeneous ensemble methods. The F1 score of majority voting was 0.8876, and that of weighted voting was 0.8885. The F1 score of the proposed 2SVB method (0.8942) is 0.0057 higher than that of the average voting (0.8885) method. The experimental results showed the advantage of our proposed ensemble method, and 2SVB performed better than all the other ensemble models.

### 4.7. Ablation Study

The ablation experiments focused on the effect of each part of the proposed 2SVB method on the ensemble’s performance. Two sets of experimental sets were set up in the ablation study. The design choices of the homogeneous ensemble mode and the heterogeneous ensemble mode were tested. The performance of 2SVB was tested by removing an individual component or a combination of them. We report the evaluation metrics for the experiments.

#### 4.7.1. Homogeneous Ensemble Modes

For the homogeneous ensemble mode, we designed the ensemble method with BERT as the base model, as shown in Table 6. Group 2 (0.8657) had a higher F1 score than group 1 (0.8641), and group 4 (0.8737) had a higher F1 score than group 3 (0.8706). This indicates the effectiveness of the stage-1 data processing using different datasets for training. The performances of groups 3 and 4 were better than those of groups 1 and 2, which indicates that our two-stage data processing and training approach could yield better results. Group 5 had the highest F1 score (0.8751), which demonstrates that 2SVB’s strategy was effective even for the homogeneous ensemble model.

#### 4.7.2. Heterogeneous Ensemble Modes

For the heterogeneous ensemble mode, we employed five PDL models, which are listed in Table 7. While the stage-2 data processing for groups 4 and 9 used normal division (3-normal), groups 5 and 10 utilized the stage-2 erroneous data to update the dataset (3-update). Group 10 (0.8942) achieved a higher F1 score than group 9 (0.8913), and group 5 outperformed group 4. This suggests that our two-stage data processing approach using erroneous data was effective. Furthermore, groups 6–10 performed better than groups 1–5, indicating that our two-stage ensemble approach using five heterogeneous models was superior to a single model. In summary, all experiments demonstrated that our two-stage strategy utilizing erroneous data and the heterogeneous pre-training framework-based model ensemble approach significantly improved the performance of 2SVB.

### 4.8. Comparison of Confusion Matrices of Base Models and the Ensemble Method

**Figure 8:**
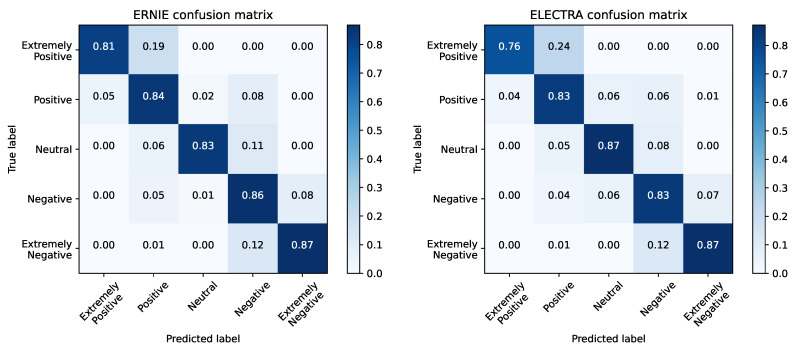

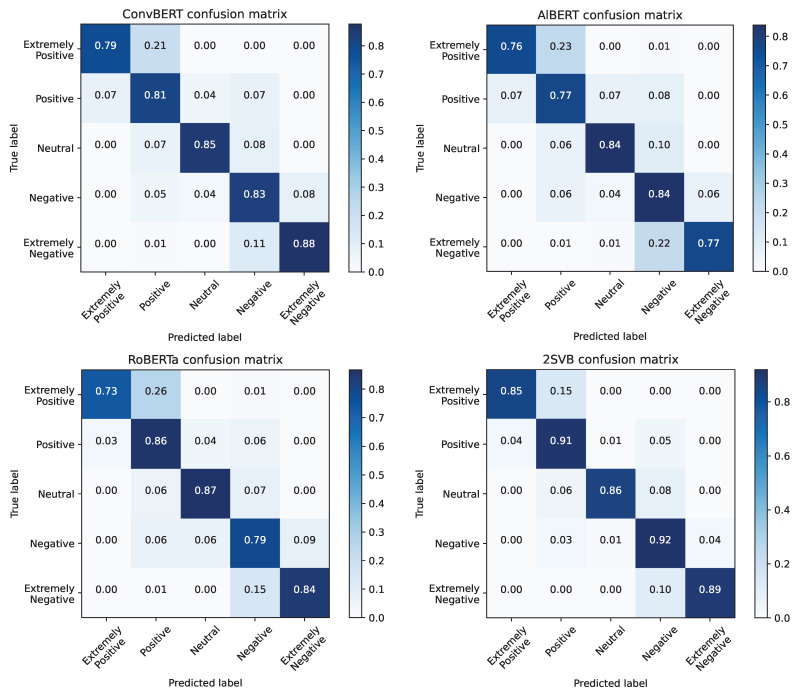
Base models and 2SVB method confusion matrix.

Herein, we compare the confusion matrices of the sentiment classification results of the five base models and the 2SVB method on the coronavirus tweets sentiment dataset, as shown in Figure 8. The confusion matrix rows represent the true labels, and columns represent the predicted labels. The right diagonal line represents the probabilities of the model’s prediction being correct, while the other positions represent the probabilities of incorrect predictions. The prediction accuracies of most of the five basic models were lower than that of the 2SVB method. However, for the probability of predicting neutral labels, 2SVB (0.86) was worse than ELECTRA (0.87) and RoBERTa (0.87). It was only 0.02, 0.01, and 0.03 more accurate than AlBERT (0.84), ConvBERT (0.85), and ERNIE (0.83). We guessed that too much erroneous data was put into the training set, which affected the prediction of neutral labels. However, overall, the 2SVB method obtained good performance. This experiment could obviously show that our 2SVB method has better performance than the base classifier on the coronavirus tweets multi-category sentiment dataset.

## 5. Conclusions

This paper proposes a novel 2SVB ensemble learning method based on the PDL model to achieve better sentiment classification of coronavirus tweets. The proposed two-stage data processing approach not only uses diverse data, but also leverages erroneous data. We constructed two-stage concurrent training and ensemble frameworks based on five PDL models with heterogeneous pre-training frameworks. All training processes, except for the collection process of erroneous data, can be computed concurrently. By utilizing heterogeneous PDL models, we increased the diversity of base classifiers of the 2SVB and further improved the ensemble performance. Additionally, we proposed a concurrent ensemble method of cascaded voting in the stage-2 ensemble, which enhances the diversity of concurrent ensemble algorithms. Our experiments demonstrated that the proposed two-stage data processing approach outperformed other data processing methods. Among the compared ensemble combinations, the combination of ERNIE2, ELECTRA, ConvBERT, AlBERT, and RoBERTa achieved the best results. The F1 score of the 2SVB method surpassed those of the other ensemble methods, demonstrating better sentiment classification performance. Furthermore, we conducted ablation experiments to evaluate the performance of 2SVB by removing a single component or a combination of them. The experimental results show that the strategy of the 2SVB multiple-use dataset and the concurrent ensemble method based on the heterogeneous PDL model could achieve superior sentiment classification of coronavirus tweets.

## Figures and Tables

**Table 1:** Results of the base models in the training stage.

Model	Training		F1	Rec	Pre	Acc
ERNIE	stage 1 training	1	0.8327	0.8431	0.8258	0.8275
		2	0.8516	0.8610	0.8464	0.8470
		3	0.8485	0.8637	0.8391	0.8447
	stage 2 training	4	0.8501	0.8608	0.8426	0.8444
		5	0.8581	0.8664	0.8519	0.8547
		6	0.8613	0.8681	0.8559	0.8555
ELECTRA	stage 1 training	1	0.8406	0.8521	0.8333	0.8328
		2	0.8496	0.8531	0.8471	0.8436
		3	0.8479	0.8568	0.8412	0.8423
	stage 2 training	4	0.8473	0.8575	0.8397	0.8418
		5	0.8455	0.8480	0.8431	0.8412
		6	0.8367	0.8443	0.8330	0.8333
ConvBERT	stage 1 training	1	0.8480	0.8521	0.8451	0.8428
		2	0.8479	0.8520	0.8445	0.8428
		3	0.8357	0.8402	0.8325	0.8310
	stage 2 training	4	0.8436	0.8518	0.8379	0.8394
		5	0.8539	0.8655	0.8451	0.8478
		6	0.8401	0.8459	0.8353	0.8344
AlBERT	stage 1 training	1	0.8024	0.8135	0.7946	0.7975
		2	0.8406	0.8538	0.8316	0.8362
		3	0.8238	0.8238	0.8249	0.8175
	stage 2 training	4	0.8267	0.8336	0.8224	0.8217
		5	0.8268	0.8233	0.8317	0.8202
		6	0.8361	0.8349	0.8392	0.8296
RoBERTa	stage 1 training	1	0.8132	0.8167	0.8122	0.8065
		2	0.8190	0.8217	0.8173	0.8125
		3	0.8057	0.8096	0.8025	0.7970
	stage 2 training	4	0.8386	0.8423	0.8366	0.8318
		5	0.8300	0.8371	0.8253	0.8239
		6	0.8228	0.8337	0.8179	0.8183

**Table 2:** Number of labels predicted by classifiers.

Classifier Groups	ERNIE2	ELECTRA	ConvBERT	AlBERT	RoBERTa	Number of Predicted Labels	1	2	3	4	5
1	✓		✓		✓	Number of samples	3259	531	8	–	–
2			✓	✓	✓		3166	616	16	–	–
3		✓	✓	✓			3243	537	18	–	–
4	✓	✓	✓				3372	413	13	–	–
5	✓	✓	✓	✓			3183	588	27	0	–
6	✓	✓	✓	✓	✓		3045	715	38	0	0

**Table 3:** Basic statistical information of the coronavirus tweet sentiment dataset.

Statistic	Neutral	Positive	Extremely Positive	Negative	Extremely Negative	Total
Train	7713	11,422	6624	9917	5481	41,157
Test	619	947	599	1041	592	3798

**Table 4:** Experimental models’ hyperparameter settings.

Model	Optimizer	Batch Size	Initial lr	Max len
Embedding	Adam	64	$1\times 10^{-3}$	256
1-D Conv	Adam	64	$1\times 10^{-3}$	256
Bi-LSTM	Adam	64	$1\times 10^{-3}$	256
PDL	AdamW	64	$5\times 10^{-5}$	256

**Table 5:** Comparison of the ensemble performance metrics for the classifier groups.

Index	Classifier Groups	F1	Rec	Pre	Acc
1	SVM, Embedding, Bi-LSTM	0.6455	0.6523	0.6399	0.6399
2	Bi-LSTM, GPT2, BERT	0.8089	0.8141	0.8046	0.8025
3	Embedding, 1-D Conv, Bi-LSTM, GPT2, BERT	0.7546	0.7671	0.7452	0.7494
4	GPT2, BERT, RoBERTa	0.8517	0.8603	0.8448	0.8454
5	ERNIE, BERT, RoBERTa	0.8643	0.8717	0.8584	0.8594
6	ERNIE, ELECTRA, ConvBERT	0.8694	0.8777	0.8630	0.8641
7	ERNIE, ELECTRA, ConvBERT, RoBERTa	0.8710	**0.8809**	0.8635	0.8657
8	ERNIE, ELECTRA, ConvBERT, AlBERT, RoBERTa	**0.8712**	0.8801	**0.8647**	**0.8657**
9	ERNIE, ELECTRA, ConvBERT, AlBERT, RoBERTa, XLNet	0.8677	0.8772	0.8602	0.8628

**Table 6:** Ablation study on homogeneous ensemble modes.

Base Model	Group	S-1 D ^1^	S-2 D ^2^	S-1 T ^3^	S-2 T ^4^	S-1 E ^5^	S-2 E ^6^	F1	Rec	Pre	Acc
BERT	1	3ND ^7^	–	BERT*3 ^9^	–	average voting	–	0.8641	0.8694	0.8596	0.8586
	2	3FD ^8^	–	BERT*3	–	average voting	–	0.8657	0.8774	0.8574	0.8612
	3	3ND	3ND	BERT*3	BERT*3	average voting	–	0.8706	0.8788	0.8641	0.8655
	4	3FD	3ND	BERT*3	BERT*3	average voting	–	0.8737	0.8867	0.8642	0.8699
	5	3FD	3UD ^10^	BERT*3	BERT*3	average voting	–	**0.8751**	**0.8870**	**0.8664**	**0.8715**

^1^ S-1 D: stage-1 data processing; ^2^ S-2 D: stage-2 data processing; ^3^ S-1 T: stage-1 training; ^4^ S-2 T: stage-2 training; ^5^ S-1 E: stage-1 ensemble; ^6^ S-2 E: stage-2 ensemble; ^7^ 3ND: 3 normal datasets divided by 2:1; ^8^ 3FD: 3 datasets divided according to a 3-fold cross-segmentation method; ^9^ BERT*3: BERT model was trained 3 times; ^10^ 3UD: 3 updated datasets were processed as two-stage data processing.

**Table 7:** Ablation study on homogeneous ensemble modes.

Base Model	Group	S-1 D	S-2 D	S-1 T	S-2 T	S-1 E	S-2 E	F1	Rec	Pre	Acc
ERNIE ELECTRA ConvBERT AlBERT RoBERTa	6	3ND	–	every*3 ^1^	–	average voting	Cascade voting	0.8820	0.8940	0.8734	0.8786
	7	3FD	–	every*3	–	average voting	Cascade voting	0.8866	0.8945	0.8806	0.8826
	8	3ND	3ND	every*3	every*3	average voting	Cascade voting	0.8885	0.8990	0.8806	0.8849
	9	3FD	3ND	every*3	every*3	average voting	Cascade voting	0.8913	0.9028	0.8829	0.8878
	10	3FD	3UD	every*3	every*3	average voting	Cascade voting	**0.8942**	**0.9063**	**0.8853**	**0.8910**

^1^ every*3: each base model was trained 3 times.

## Data Availability

The coronavirus tweets natural language processing text classification data used to support the findings of this study are open source on the Kaggle competition platform and can be found at https://www.kaggle.com/datasets/datatattle/covid-19-nlp-text-classification (accessed on 1 June 2022).

## Abbreviations

The following abbreviations are used in this manuscript:

S-1 D	stage-1 data processing
S-2 D	stage-2 data processing
S-1 T	stage-1 training
S-2 T	stage-2 training
S-1 E	stage-1 ensemble
S-2 E	stage-2 ensemble
3ND	3 normal datasets divided by 2:1
3FD	3 datasets divided according to a 3-fold cross-segmentation method
3UD	3 updated datasets were processed as two-stage data processing
